# Two Arginine Residues of *Streptococcus gordonii* Sialic Acid-Binding Adhesin Hsa Are Essential for Interaction to Host Cell Receptors

**DOI:** 10.1371/journal.pone.0154098

**Published:** 2016-04-21

**Authors:** Yumiko Urano-Tashiro, Yukihiro Takahashi, Riyo Oguchi, Kiyoshi Konishi

**Affiliations:** 1 Department of Microbiology, Nippon Dental University School of Life Dentistry at Tokyo, Fujimi, Chiyoda-ku, Tokyo, Japan; 2 Department of Pediatric Dentistry^2^, Nippon Dental University School of Life Dentistry at Tokyo, Fujimi, Chiyoda-ku, Tokyo, Japan; Oregon Health & Science University, UNITED STATES

## Abstract

Hsa is a large, serine-rich protein of *Streptococcus gordonii* DL1 that mediates binding to α2-3-linked sialic acid termini of glycoproteins, including platelet glycoprotein Ibα, and erythrocyte membrane protein glycophorin A, and band 3. The binding of Hsa to platelet glycoprotein Ibα contributes to the pathogenesis of infective endocarditis. This interaction appears to be mediated by a second non-repetitive region (NR2) of Hsa. However, the molecular details of the interaction between the Hsa NR2 region and these glycoproteins are not well understood. In the present study, we identified the amino acid residues of the Hsa NR2 region that are involved in sialic acid recognition. To identify the sialic acid-binding site of Hsa NR2 region, we prepared various mutants of Hsa NR2 fused with glutathione transferase. Fusion proteins harboring Arg340 to Asn (R340N) or Arg365 to Asn (R365N) substitutions in the NR2 domain exhibited significantly reduced binding to human erythrocytes and platelets. A sugar-binding assay showed that these mutant proteins abolished binding to α2-3-linked sialic acid. Furthermore, we established *S*. *gordonii* DL1 derivatives that encoded the corresponding Hsa mutant protein. In whole-cell assays, these mutant strains showed significant reductions in hemagglutination, in platelet aggregation, and in adhesion to human leukocytes. These results indicate that the Arg340 and Arg365 residues of Hsa play an important role in the binding of Hsa to α2-3-linked sialic acid-containing glycoproteins.

## Introduction

The oral streptococci, including *Streptococcus gordonii*, is associated with primary dental plaque formation [1]. *S*. *gordonii* and related species of the viridans group of streptococci colonize damaged heart valves and are recognized as etiological bacterial agents of infective endocarditis (IE) [2–4].

*S*. *gordonii* adhere to saliva-coated hydroxyapatite, an experimental model of the tooth surface, and bind to host cells such as erythrocytes, platelets, polymorphonuclear leukocytes (PMNs), macrophages, and monocytes [5–10]. A common mechanism for these interactions is the recognition of surface-associated host sialoglycoconjugates. The binding of *S*. *gordonii* to human platelets on the surface of damaged cardiac valves is an important initiation step in the pathogenesis of IE [11]. Recent studies have reported the adhesins of streptococci that bind human platelets including serine-rich surface proteins designated Hsa, GspB, SrpA, and Srr1, an antigen I/II (AgI/II) family polypeptides SspA and SspB, and surface proteins PadA, PblA, and PblB [12–18]. Attachment of *S*. *gordonii* DL1 to host cells is facilitated predominantly by Hsa, which mediates bacterial binding to the sialic acid moiety of receptors on host cell membrane.

*S*. *gordonii* DL1 Hsa consists of an N-terminal nonrepetitive region (NR1), a serine-rich region (SR1), another nonrepetitive region (NR2), another serine-rich region (SR2), and a C-terminal cell wall-anchoring region [19]. Hsa binds to the α2-3-linked sialic acid-containing glycoptoteins, including salivary mucin MG2, platelet glycoprotein Ibα (GPIbα), and leukosialin (CD43), the major surface protein of human PMNs [5, 12, 20, 21]. The NR2 region of Hsa is involved in the binding interaction between α2-3-linked sialic acid and Hsa [7]. In previous work, we identified glycophorin A (GPA) and band 3 as erythrocyte receptors for Hsa [8]. Moreover, CD11b and CD50 have been identified as leukocyte receptors for Hsa [9]. Two Hsa homologues, GspB of *S*. *gordonii* strain M99 and SrpA of *Streptococcus sanguinis* strain SK36, bind human platelets through sialic acid-dependent interactions with GPIbα [14, 22].

Hsa, GspB, and SrpA are all α2-3-linked sialic acid-binding proteins, but the three homologues show distinct specificities. The NR2 of Hsa and the binding region of SrpA each can bind both 3’-sialyllactose [Neu5Acα2-3Gal^®^1-4Glc] and sialyl-T antigen [Neu5Acα2-3Gal^®^1-3GalNAc], whereas the binding region of GspB binds only sialyl-T antigen [13, 23]. The binding region of GspB contains the V-set Ig fold adopted by eukaryotic Siglec (sialic acid binding immunoglobulin-like lectins)-like domain. Moreover, Arg484 of GspB is important for binding to α2–3 sialyl-glycoproteins [24]. Hsa contains a Siglec-like domain in NR2 [24]. However, the molecular details of the interaction between Hsa NR2 region and the target sialylated carbohydrates are not well understood. In the present study, we have identified two amino acid residues of Hsa that are involved in sialic acid interaction. Our results show that Arg340 and Arg365 of Hsa play an important role in the binding of *S*. *gordonii* DL1 to human cell surface glycoproteins.

## Materials and Methods

### Ethics statement

All research involving human participants have been approved by our Instructional Review Board. Healthy donor was informed and gave his written consent for using the collected samples in a scientific study. Collection and use of blood samples in this study was approved by the Research Ethics Committee of Nippon Dental University (NDU-T2012-33).

### Bacterial strains and growth conditions

The *S*. *gordonii* strains used in this study were DL1 (Challis strain; wild type) and its derivative CM100 (DL1 ⓧ*hsa cat*) [6, 7]. Streptococci were cultured overnight at 37°C in brain heart infusion broth (Becton Dickinson, Franklin Lakes, NJ). The medium was supplemented (as needed) with 200 μg/ml of spectinomycin dihydrochloride or 8 μg/ml of chloramphenicol (Sigma-Aldrich, St. Louis, MO). *Escherichia coli* BL21 harboring the plasmids with ampicillin resistance was grown in Luria-Bertani broth containing 100 μg/ml ampicillin (Sigma-Aldrich).

### Construction and purification of glutathione *S*-transferase-HsaNR2 fusion proteins.

Glutathione *S*-transferase (GST) -HsaNR2 fusion protein was constructed, and the expressed protein was purified, as described previously [8]. Site-directed mutagenesis was performed using the QuickChange Site-Directed Mutagenesis kit (Strategene, La Jolla, CA) and the following primer pairs: R308Q (Arg308 to Gln308) (5’-primer: 5’-GAG AAT CTT GGT CAG CCT GGT AAT GC-3’, 3’-primer: 5’-GCA TTA CCA GGC TGA CCA AGA TTC TC-3’), R319N (5’-primer: 5’-CCT CTC AAC ACT AGA ATA TTT GGT G-3’, 3’-primer: 5’-CAC CAA ATA TTC TAG TGT TGA GAG G-3’), R321N (5’-primer: 5’-CCT CTC CGG ACT AAC ATA TTT GGT G-3’, 3’-primer: 5’-CAC CAA ATA TGT TAG TCC GGA GAG G-3’), R340N (5’-primer: 5’- CTT ATT ATA CTA ATT ATA TAG TTG CG -3’, 3’-primer: 5’- CGC AAC TAT ATA ATT AGT ATA ATA AG -3’), R360N (5’-primer: 5’-GGA TAA TGC TAA TAA CAA TGG ACT AG-3’, 3’-primer: 5’-CTA GTC CAT TGT TAT TAG CAT TAT CC-3’), and R365N (5’-primer: 5’- GAA TGG ACT AGA AAA CTT TGT CC -3’, 3’-primer: 5’- GGA CAA AGT TTT CTA GTC CAT TC -3’). The plasmids containing genes encoding HsaNR2 with R308Q, R319N, R321N, R340N, R360N, or R365N mutations were designated as pGEX-HsaNR2R308Q, pGEX-HsaNR2R319N, pGEX-HsaNR2R321N, pGEX-HsaNR2R340N, pGEX-HsaNR2R360N, and pGEX-HsaNR2R365N, respectively.

### Hemagglutination assay

The hemagglutination (HA) assay was performed as previously described [6] by mixing 25 μl of serial dilutions of bacteria (starting at 2 × 10^9^ cells/ml) or GST-fusion protein (starting at 2 mg/ml) with equal volumes of 1% human type-O erythrocytes in the individual wells of 96-well round-bottom microtiter plates. The plates were incubated at 4°C overnight. Positive results were scored as agglutinated cell sheets compared to non-agglutinated cell bottoms. Images of hemagglutination were taken with a digital camera (EOS X3, Canon, Japan).

### Platelet aggregation assay

The platelet bacterial adhesion assay was performed as previously described [7] using platelets obtained by centrifugation of platelet-rich plasma from citrated human type O blood. Specifically, aliquots (50 μl) of bacteria (at 2 × 10^9^ cells/ml) or GST-fusion protein (at 2 mg/ml) were mixed with equal volumes of washed platelets (at 5 × 10^8^ cells/ml) in the individual wells of 96-well microtiter plates. The plates were incubated for 30 min at room temperature (RT). Supernantants containing unaggregated platelets were harvested following low-speed centrifugation (35 × *g*) and were diluted 1:2 with Tris-buffered saline (TBS; 20 mM Tris-HCl, pH 7.8, 150 mM NaCl, 10 mM ethylenediaminetetraacetic acid) prior to measurement of absorbance at 620 nm (*A*_620_). Differences in platelet aggregation were statistically analyzed using unpaired *t*-test. *P* values < 0.05 were considered statistically significant.

### Sugar-binding assay

GST-HsaNR2 fusion proteins were diluted to 500 nM into phosphate-buffered saline (PBS) and then subjected to serial two-fold dilutions in PBS. 50 μl of each dilution were transferred to the individual wells of a 96-well microtiter plate. After adsorption by overnight incubation at RT, wells were rinsed with PBS and blocked with PBS containing 1% blocking reagent (Roche, Mannheim, Germany) for 1 h at RT. The wells were incubated for 90 min at RT with 50 μl of biotinylated 3’-sialyllactose or sialyl-T antigen (each at 500 ng/ml; GlycoTech, Gaithersburg, MD), and unbound biotinylated 3’-sialyllactose and sialyl-T antigen was removed by rinsing three times with PBS. 50 μl of avidin-_D_-horseradish peroxidase (0.1 μg/ml in PBS) (Vector, Burlingame, CA) was added to each well; plates were incubated for 1 h at RT, and the unbound peroxidase was removed by rinsing three times with PBS. An aliquot (100 μl) of a solution of *o*-phenylenediamine (OPD; 1 mg/ml in citrate-phosphate buffer) was added to each well; following incubation for several minutes at RT, 50 μl of 2 M H_2_SO_4_ was added to each well to stop the reaction. The contents of the wells were mixed by gently vortexing the plate, and *A*_490_ was measured. Data are provided as the mean ± standard deviation, with n = 6.

### Generation of *hsa* NR2 point mutant constructs

Each *hsa* NR2 point mutant plasmid was prepared from plasmid pAS8743, which lacks a BglII site within the multiple cloning site of pAS8741 [7]. HsaNR2 mutants substituting Arg340 and Arg365 for Asn were created by PCR using the QuickChange Site-Directed Mutagenesis kit and the primers described above. pIRH206, a deletion mutant derivative of pIRH801 harboring a 1.9-kb fragment [19], was used as the template for these PCR reactions. pAS8743R340N was constructed by the insertion of the HindIII-BglII fragment from pIRH206R340N into the HindIII-BglII site of pAS8743. pAS8743R365N was constructed by the insertion of the HindII-BglII fragment from pIRH206R365N into the HindIII-BglII site of pAS8743. These plasmids were transformed as previously described [25] into *S*. *gordonii* CM100. The transformants harboring the original and mutated plasmids (pAS8743WT, pAS8743R340N, and pAS8743R365N) were designated CM100 (WT), CM100 (R340N), and CM100 (R365N), respectively. Expression of mutant Hsa proteins on the bacterial cell surface was verified by western blotting as described below.

### Analysis of cell wall proteins

Bacteria were harvested from cultures in the logarithmic phase of growth. 1 × 10^10^ of bacterial cells were digested with mutanolysin (Sigma-Aldrich) in the presence of raffinose (26% wt/vol), as described previously [7]. Samples were subjected to sodium dodecyl sulfate-polyacrylamide gel electrophoresis (SDS-PAGE) on a 3 to 8% gradient gel (Life Technologies, Carlsbad, CA), and electrophoretically transferred to PVDF membranes (Bio-Rad, Hercules, CA) for western blotting with anti-Hsa (1:1000 dilution) or anti-DL1 (1:10,000 dilution) antibody as previously described [7].

### Attachment of bacteria to human leukocytes

Human leukocytes were isolated from peripheral blood collected from healthy donors. Erythrocytes were removed by dextran segmentation and hypotonic lysis. Leukocytes were washed three times and resuspended in RPMI-1640 supplemented with 1% bovine serum albumin (BSA), 0.2% HEPES, and 0.15 mM CaCl_2_. Leukocyte preparations (1×10^6^ cells/ml) were challenged with bacteria (1×10^8^ cells/ml) for 2 h at 37°C. Cells were washed twice with 1 × PBS containing 1% BSA and stained by the Wright-Giemsa method using Diff-Quick (sysmex, Kobe, Japan). Bacteria binding to leukocytes were quantitatively evaluated by counting the bacterial cell number on the surface of 30 randomly selected neutrophils, eosinophils, monocytes, or lymphocytes. The glass slides were imaged by a upright microscope (LABOPHOT-2, Nicon, Japan). Differences between means were compared for statistical significance by the unpaired *t*-test using *P* < 0.05 as the threshold for significance.

## Results

### The middle of the Hsa NR2 region is required for interaction with erythrocytes

**Fig 1:**
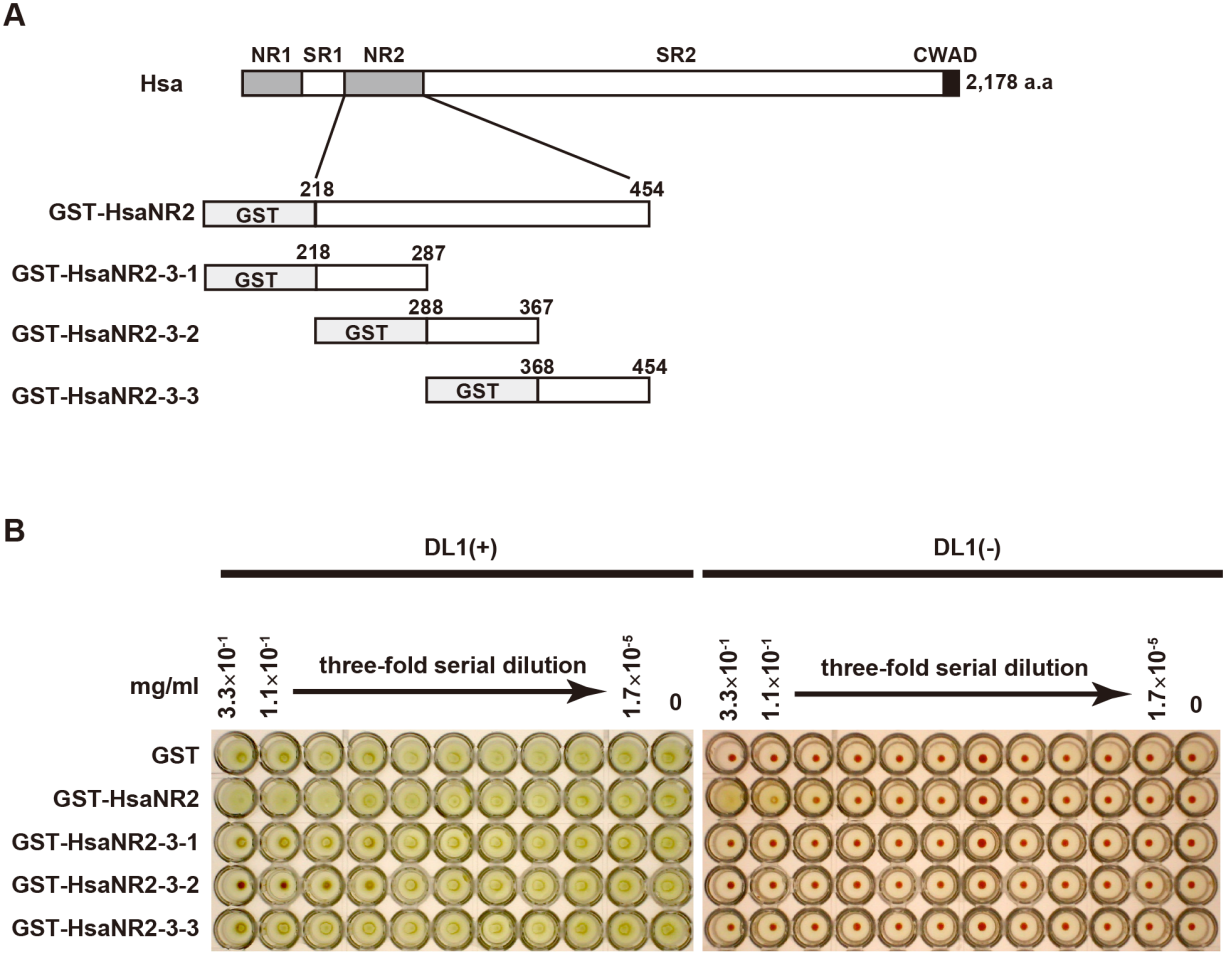
GST-HsaNR2-3-2 inhibits hemagglutination of human erythrocytes by *S*. *gordonii* DL1. (A) Schematic drawing of GST fusion proteins. NR1 and NR2, non-repetitive regions 1 and 2 (respectively); SR1 and SR2, serine-rich repeat regions 1 and 2 (respectively); CWAD, cell wall-anchoring domain. (B) Hemagglutination of human erythrocytes by *S*. *gordonii* DL1. 0.3% human erythrocytes were incubated with serial dilutions of GST-fused HsaNR2 mutant proteins in the presence or absence of *S*. *gordonii* DL1.

To define the region of Hsa required for the interaction with sialic acid, we prepared three truncation mutants of HsaNR2 fused with GST (Fig 1A). We previously reported that Hsa bound to erythrocyte membrane-associated protein in a α2-3-linked sialic acid-dependent manner [8]. In the present work, we used the HA assay to test the ability of GST-fused protein to bind α2-3-linked sialic acid. Erythrocytes were pretreated with each of several GST-fused proteins, followed by the bacterial HA assay. Bacteria-mediated HA was inhibited only by GST-HsaNR2-3-2 (Fig 1B left panel), a construct that includes Hsa residues corresponding to amino acids 298–377 of the full-length protein. Other GST-fused proteins had no effect on bacteria-mediated HA. In the control experiment, HA was observed when erythrocytes were incubated with GST-HsaNR2 (Fig 1B right panel) alone, as described previously [8]. This result indicates that the 298–377 region of Hsa binds to α2-3-linked sialic acid-containing erythrocyte surface glycoproteins.

### Hemagglutination activity is abolished by conservative substitution of a single Arg residue

**Fig 2:**
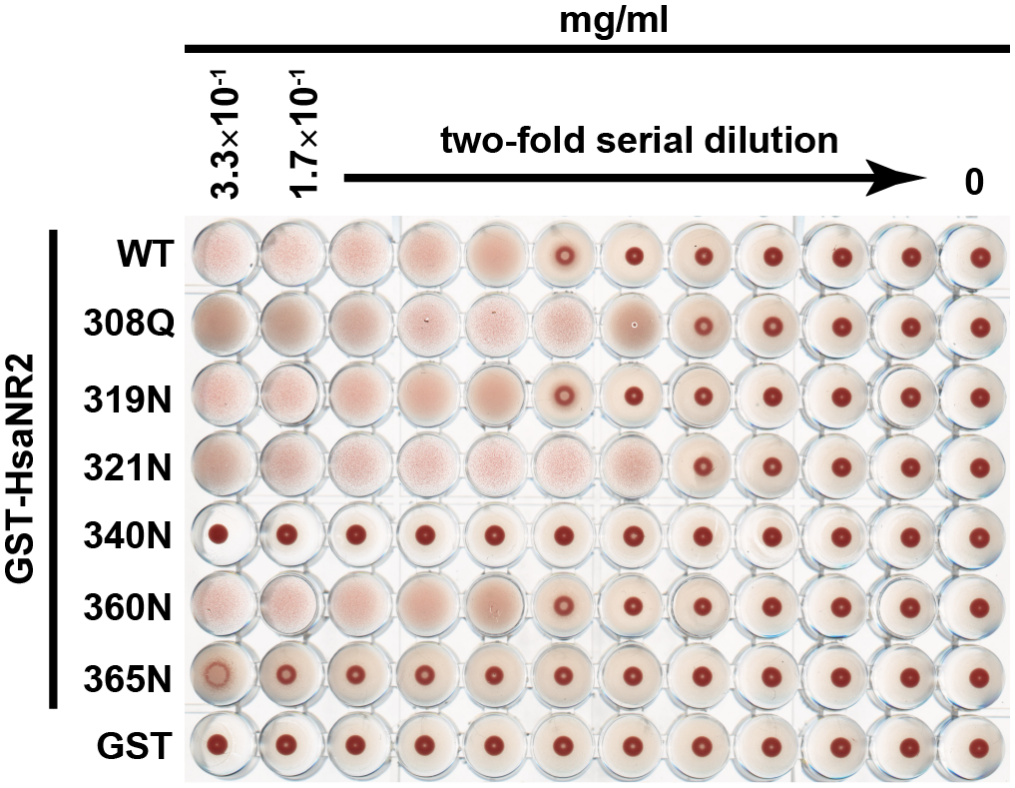
Hemagglutination of human erythrocytes by GST-HsaNR2 mutant proteins. 0.3% human erythrocytes were incubated (overnight at 4°C) with serial dilutions of GST-fused HsaNR2 mutant proteins.

To identify the amino acid residue(s) of the Hsa 298–377 region involved in α2-3-linked sialic acid recognition, we constructed six mutants of GST-HsaNR2, each of which contained a single substitution at an arginine residue (R308Q, R319N, R321N, R340N, R360N, and R365N), and repeated the HA assay (Fig 2). As expected, HA was observed in a control experiment, when erythrocytes were incubated with GST-HsaNR2WT. HA activity was abolished completely when erythrocytes were incubated with GST-HsaNR2R340N or GST-HsaNR2R365N. Other mutant GST-HsaNR2 proteins (with R308Q, R319N, R321N, or R360N lesions) had HA activities similar to that observed with GST-HsaNR2WT. These results show that Arg340 and Arg365 play a role in the interaction between Hsa and α2-3-linked sialic acid-containing glycoproteins.

### Arg 340 and Arg365 are essential for Hsa adhesion to human platelets

**Fig 3:**
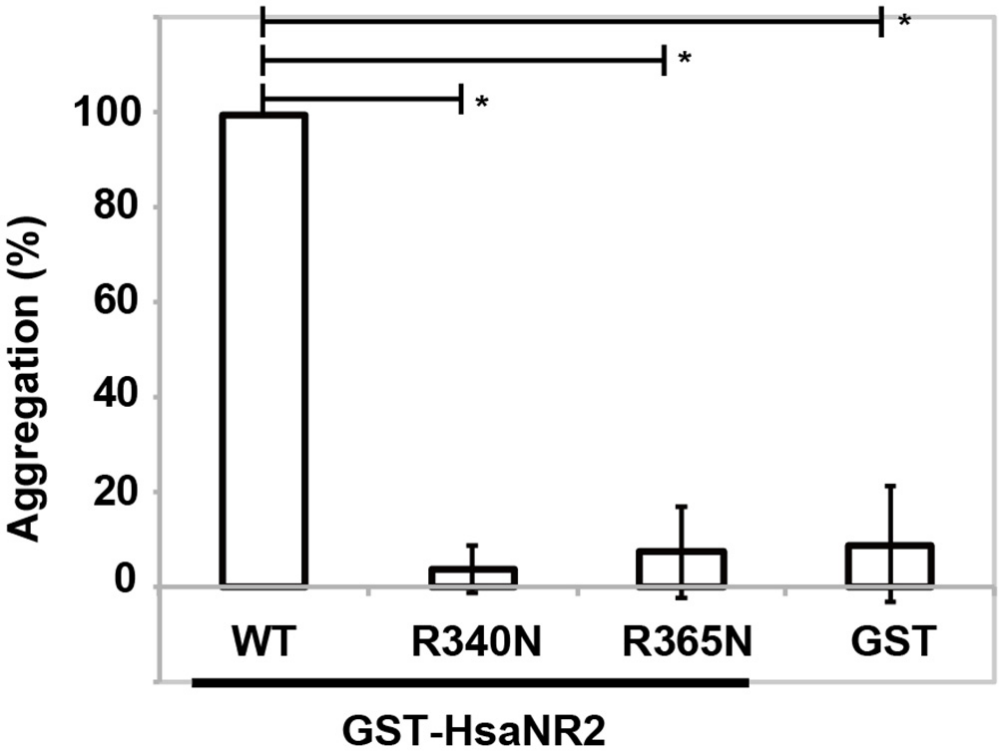
Mutation of Arg340 and Arg365 residues abolishes platelet aggregation activity. Washed human platelets were incubated (for 30 minutes at room temperature) with 0.33 mg/ml of GST, GST-HsaNR2WT, or GST-HsaNR2 proteins harboring R340N or R365N substitutions. Percent aggregation of platelets was determined spectrophotometrically. The mean numbers of percent aggregation and standard deviations were determined by six independent experiments. Statistical differences in the means of obtained values were evaluated by an unpaired *t*-test (* *P* < 10^-5^).

We selected the R340N and R365N mutations for more detailed evaluation. *S*. *gordonii* DL1 has not only HA activity but also platelet aggregation activity. Therefore, we analyzed the involvement of Arg340 and Arg365 in the attachment of Hsa to platelets. Washed human platelets were mixed with GST-HsaNR2 mutant proteins for 30 min at room temperature (Fig 3). Almost full aggregation was observed when GST-HsaNR2WT was mixed with platelets (99.4 ± 0.28%). In contrast, GST-HsaNR2R340N, GST-HsaNR2R365N, and GST exhibited reduced interactions with platelets in the same assay (3.70 ± 5.11%, 7.36 ± 9.66%, and 8.90 ± 12.3%, respectively). These results demonstrate that Arg340 and Arg365 are involved in the binding of Hsa to platelets.

### Arg340 and Arg365 are involved in binding to α2-3-linked sialylated carbohydrates

**Fig 4:**
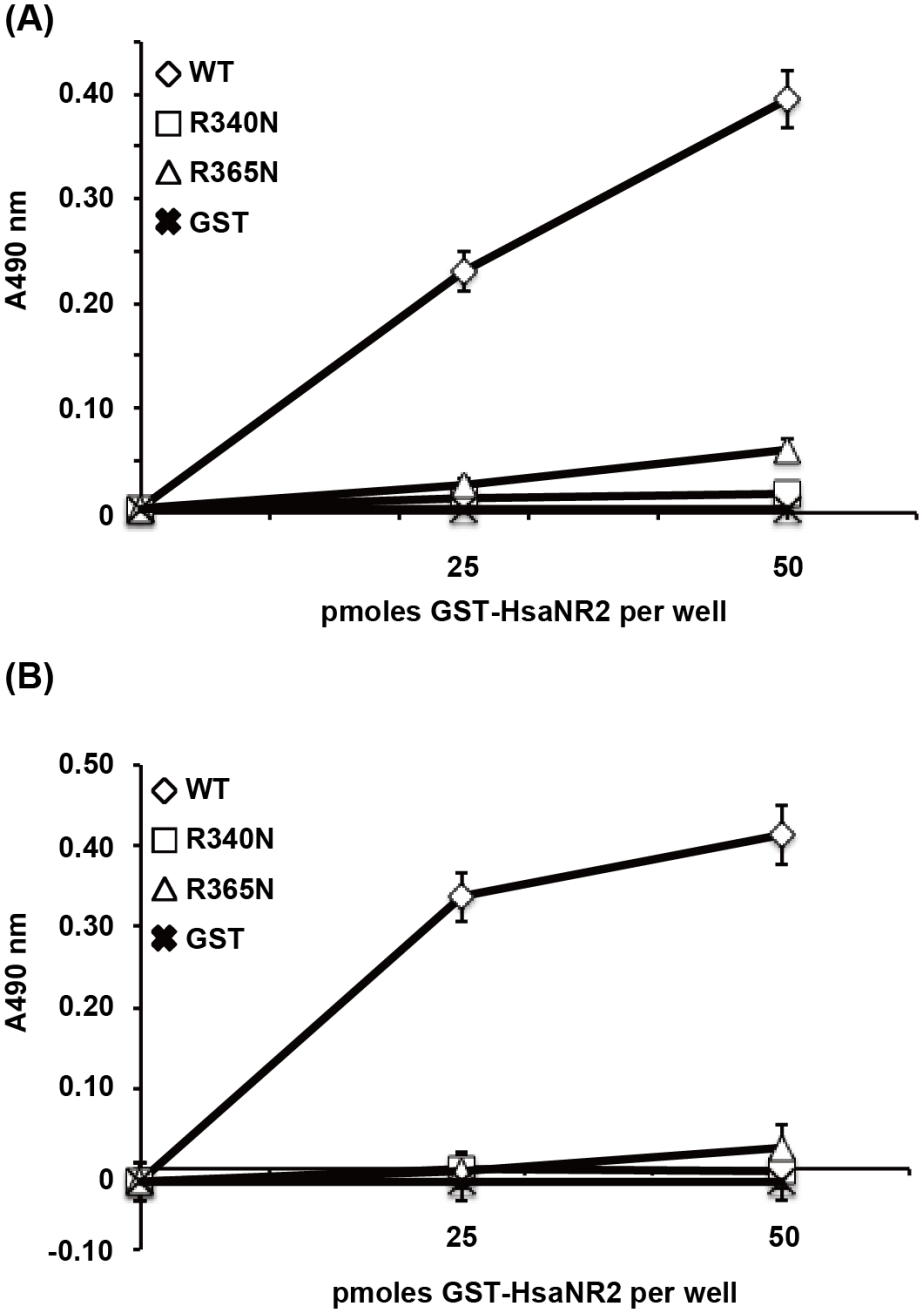
Binding of GST-HsaNR2 mutant proteins to different oligosaccharide structures. The indicated amount of GST or GST-HsaNR2 wild type (WT) or mutant (R340N and R365N) proteins were immobilized in microtiter wells, and the binding of biotinylated Neu5Acα2-3Gal^®^1-4GlcNAc (3’-sialyllactose) (A) and Neu5Acα2-3Gal^®^1-3GalNAc (sialyl-T antigen) (B) to each protein was detected using peroxidase-conjugated streptavidin along with a chromogenic peroxidase substrate. Binding is expressed as the mean ± standard deviation from the results of six independent experiments.

Hsa can bind not only to 3’-sialyllactose but also to sialyl-T antigen [13]. Therefore, we examined the sialic acid-binding specificity of GST-HsaNR2 mutant proteins using ELISA assay. GST-HsaNR2WT bound both 3’-sialyllactose and sialyl-T antigen, whereas GST-HsaNR2R340N and GST-HsaNR2R365N completely eliminated the binding of any trisaccharide structures (Fig 4). Binding activity of either of the two mutant proteins was comparable to that of GST alone, a negative control. These results indicate that Arg340 and Arg365 are necessary for sialic acid binding activity of Hsa.

### Arg340 and Arg365 are required for the sialic acid binding activity of *S*. *gordonii* DL1

**Fig 5:**
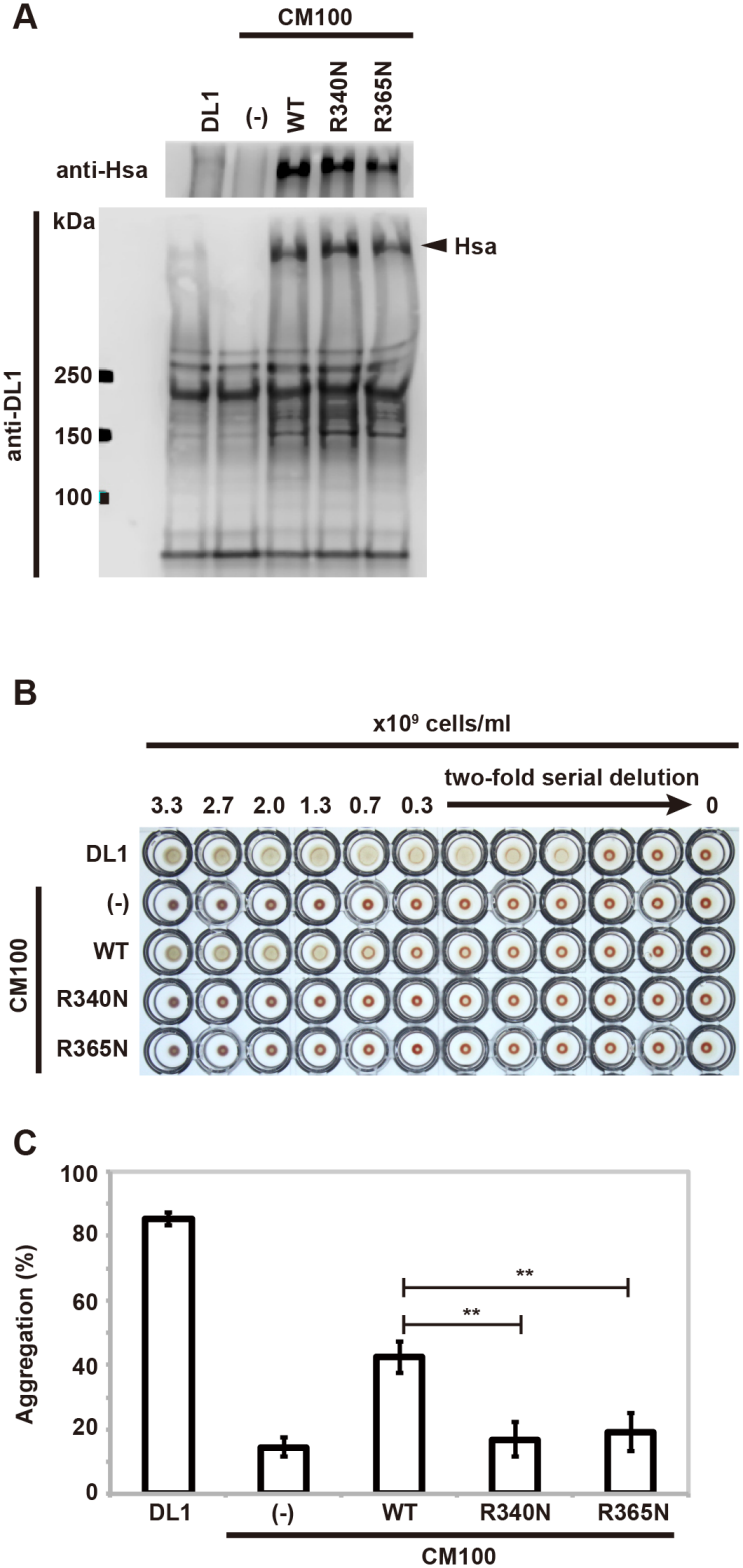
Essential role of Hsa Arg340 and Arg365 in adhesion to erythrocytes and platelets. (A) Expression of wild-type and mutant Hsa proteins on the *S*. *gordonii* cell surface. Peptidoglycan-linked proteins were extracted from bacterial cell surface and analyzed by western blotting using anti-Hsa or anti-DL1 antibody. The position of the molecular mass marker is indicated on the left in kilodaltons. (B) Hemagglutination of human erythrocytes by *S*. *gordonii*. 0.3% human erythrocytes were incubated (overnight at 4°C) with serial dilutions of *S*. *gordonii* DL1 or derivative strains. (C) Platelet aggregation activity of *S*. *gordonii*. Washed human platelets were incubated (for 30 minutes at room temperature) with *S*. *gordonii* DL1 or derivative strains. Percent aggregation of platelets was determined spectrophotometrically. The mean numbers of percent aggregation and standard deviations were determined by five independent experiments. Statistical differences in the means of obtained values were evaluated by an unpaired *t*-test (**P < 0.0001).

To confirm further that Arg340 and Arg365 of Hsa are required for the binding of α2-3-linked sialic acid-containing glycoproteins, we established *hsa* mutants of *S*. *gordonii* DL1 and assayed the resulting strains in whole-cell assays. Plasmids harboring mutated *hsa* were prepared from pAS8743WT and transformed into *S*. *gordonii* CM100, an *hsa*-deleted strain. The transformants harboring these plasmids (pAS8743WT, pAS8743R340N, and pAS8743R365N) were designated CM100 (WT), CM100 (R340N), and CM100 (R365N), respectively. The amount of Hsa on the bacterial cell surface was analyzed by western blotting with anti-Hsa antibody. None of the point mutations affected surface expression of Hsa. Western blot profile with anti-DL1 panel showed that the proteins except Hsa were expressed at equivalent levels in all the mutations and wild-type cells (Fig 5A). We next analyzed the HA activity of *S*. *gordonii* CM100-derived strains. *S*. *gordonii* DL1 and CM100(WT) had HA activity, while CM100 (R340N), CM100 (R365N), and CM100 (with no plasmid) lacked HA activity (Fig 5B). The platelet aggregation assay revealed that a large number of platelets were aggregated with *S*. *gordonii* DL1 (85.4 ± 2.10%) and CM100 (WT) (42.3 ± 4.78%). In contrast, platelet aggregation was decreased with CM100 (R340N) (16.9 ± 5.42%), CM100 (R365N) (19.3 ± 5.98%), and CM100 (14.5 ± 3.08%) (Fig 5C). These results show the importance of the Arg340 and Arg365 residues for the binding of *S*. *gordonii* DL1 to erythrocyte and platelet surface glycoproteins.

### Adhesion of *S*. *gordonii* DL1 to human leukocytes is mediated by Arg340 and Arg365 of Hsa

**Fig 6:**
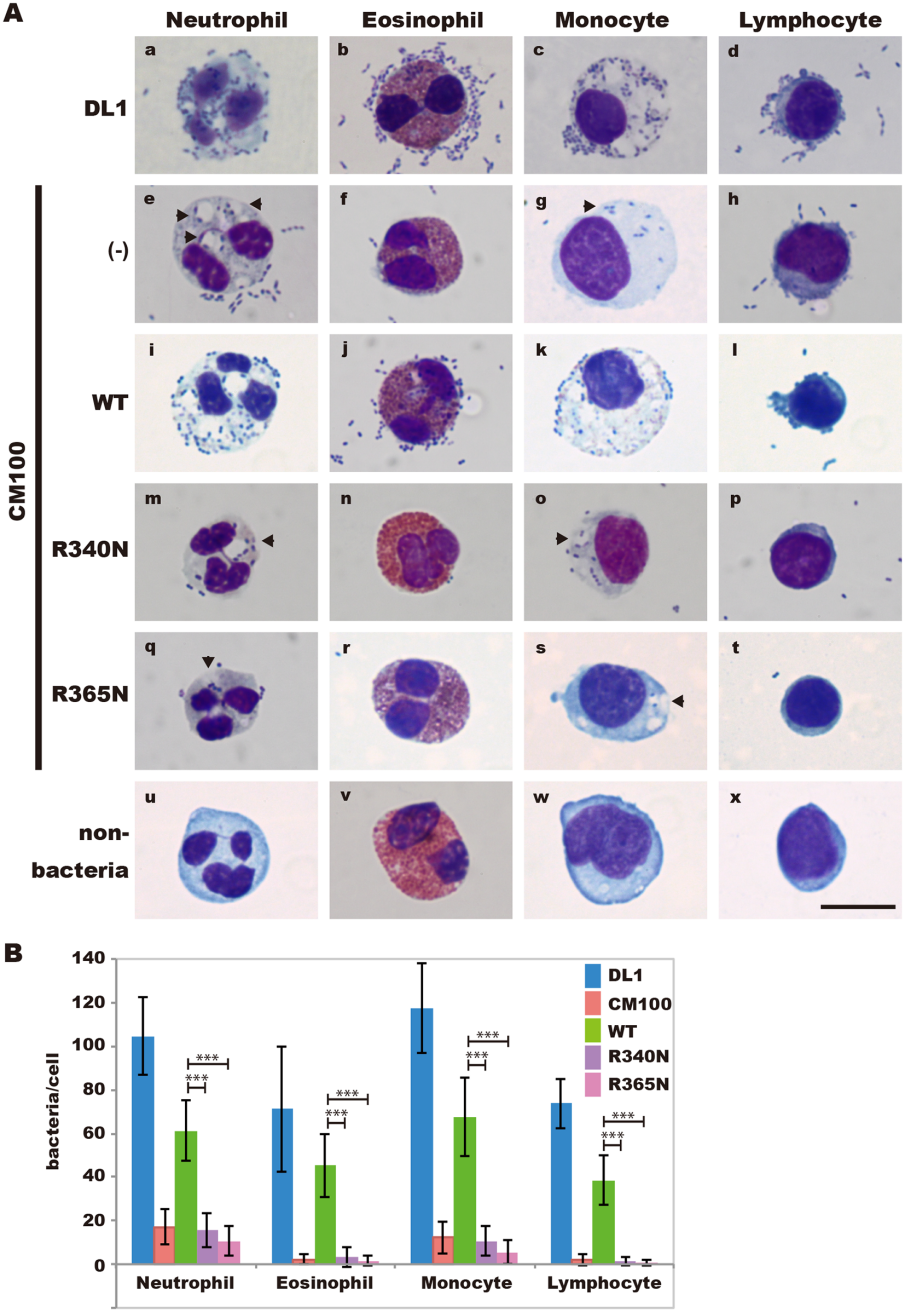
Attachment of *S*. *gordonii* DL1 to human leukocytes. (A) Human leukocytes (5× 10^5^ cells) were incubated with *S*. *gordonii* DL1 (a, b, c, and d), CM100 (e, f, g, and h), CM100 (WT) (i, j, k, and l), CM100 (R340N) (m, n, o, and p), or CM100 (R365N) (g, r, s, and t) (5× 10^7^ cells) for 2 h at 37°C. Bacterial binding was determined microscopically using Wright-Giemsa staining. Arrowheads indicate phagosomes that appear to contain intact bacterial cells. Original magnification, × 1000. Scale bar = 10 μm. (B) The means and standard deviations of bacteria cell numbers bound to randomly selected neutrophils, eosinophils, monocytes, or lymphocytes (n = 30) are indicated. Statistical differences in the means of obtained values were evaluated by an unpaired *t*-test (*** *P* < 10^-14^).

We reported previously that the binding of *S*. *gordonii* DL1 to monocytes, granulocytes, and macrophages is mediated by Hsa [9]. Therefore we examined the ability of *S*. *gordonii* CM100 strains to adhere to human peripheral leukocytes, such as neutrophils, eosinophils, monocytes, and lymphocytes. Human leukocytes were incubated for 2 h with *S*. *gordonii* strains at a ratio of 100 bacterial cells per host cell. Large numbers of bacterial cell adhered to host cells incubated with *S*. *gordonii* DL1 (Fig 6Aa–6Ad) or CM100 (WT) (Fig 6Ai–6Al). By contrast, when CM100 (Fig 6Ae–6Ah), CM100 (R340N) (Fig 6Am–6Ap), or CM100 (R365N) (Fig 6Aq–6At) were incubated with host cells, low numbers of bacterial cells bound to host cells. All of these differences were statistically significant (*P* < 10^-14^) (Fig 6B). These results indicate that Arg340 and Arg365 of Hsa play an essential role in bacterium-host cell interaction.

## Discussion

Hsa is a cell surface protein of *S*. *gordonii* that binds to the α2-3-linked sialic acids contained in glycoproteins. The binding of Hsa to sialic acid is mediated by the NR2 region of Hsa. Some glycoproteins, including GPIbα, GPA, and band 3, have been identified as receptors for Hsa. However, the molecular details of the interaction between Hsa NR2 region and these glycoproteins are not well understood. To determine the amino acid residues that mediate the interaction between Hsa and sialic acid, we used GST-fused proteins.

The results of our examination demonstrate that Arg340 and Arg365 in the NR2 region are critical for the sialic acid-binding activity of Hsa. Mutation of these amino acid residues abrogated the ability of both purified protein and bacterial cells to interact with host cells.

This study indicated that Hsa can bind a broad range of α2-3-linked sialic acids, including 3’-sialyllactose, sialyl-T antigen, and Neu5Acα2-3Gal^®^1-4GlcNAc. These results are consistent with the previous study [23]. GST-tagged NR2 domains harboring mutations at either Arg340 or Arg365 exhibited significantly reduced binding to each of the tested sialic acids. Structural prediction using Spanner server (https://sysimm.ifrec.osaka-u.ac.jp/spanner/) suggests that the middle of the NR2 region of Hsa, including Arg340 and Arg365, forms a pocket. These two Arg are predicted to be positioned on opposite sides of the hypothesized pocket. Thus, the bound sialic acid may be bracketed by Arg340 and Arg365 upon binding by Hsa. In GspB, Arg484 is required for binding to sialyl-T antigen [24]. Amino acid sequence alignment suggests that Arg484 of GspB corresponds to Arg340 of Hsa [13]. Arg365 of Hsa corresponds to Lys509 of GspB. As Lys is a positively charged amino acid, this residue should show affinity for a negatively charged sialic acid. Indeed, we found that a mutant protein in which Arg365 is changed to Lys retains weak sialic acid binding activity (data not shown). This Arg residue is conserved among many Hsa homologues reported in public databases and in sequence data from our laboratory (data not shown). Thus, this Arg is predicted to be critical to the binding of sialic acid by other streptococci.

*S*. *gordonii* binds to host blood cells such as erythrocytes, platelets, PMNs, and monocytes [5, 7–9, 12]. That binding is attributed to interactions between Hsa and the α2-3-linked sialic acid glycoproteins that are displayed on the host cell surface. In extension of our GST-HsaNR2 studies, we established derivatives of a *S*. *gordonii* DL1 *hsa* mutant strain that expressed HsaR340N or HsaR365N. Expression of the mutant protein dramatically decreased whole-cell binding of *S*. *gordonii* to erythrocytes [26] and to leukocytes (e.g., neutrophils, eosinophils, monocytes, and lymphocytes). These results indicate that Arg340 and Arg365 are involved in the interaction between *S*. *gordonii* and host cell sialoglycoproteins.

The first step in the pathogenesis of IE is the deposition of platelet-fibrin aggregates on injured valves. Bacteria circulating in the blood bind to such nonbacterial thrombotic endocarditic lesions [27]. Platelet aggregation by *S*. *gordonii* CM derivatives CM100 (R340N) and CM100 (R365N) was significantly reduced. Thus, Arg340 and Arg365 of Hsa are important for binding to platelets. This result suggests that Arg340 and Arg365 of Hsa contribute to the pathogenesis of IE by *S*. *gordonii* DL1.

IE is a serious septic disease. After the formation of vegetation on the damaged endothelium, microbial shedding by continuous bacteremia and embolization of the vegetation fragment can lead to multiple organ dysfunction syndromes such as ischemic stroke, brain abscess, and mycotic aneurysm [27]. Despite advances in diagnosis, antibacterial therapy, and surgical techniques, the incidence of and mortality from IE has not been reduced over the past 30 years. The incidence of IE is 1.4–12.7 per 100,000 person-years. Without treatment, IE is frequently lethal. One-year mortality is up to 40% [27]. Prevention of IE requires treatment with high-dose antimicrobial agents before invasive surgical techniques such as dental procedures [28]. This treatment is burden to patients. Drugs that interact with the Arg340- and Arg365-containing region of Hsa might find application as new inhibitors of bacterial binding. Our insight thus may facilitate the development of new drugs for the prevention of IE.
