# Correction: Two Arginine Residues of *Streptococcus gordonii* Sialic Acid-Binding Adhesin Hsa Are Essential for Interaction to Host Cell Receptors

**DOI:** 10.1371/journal.pone.0161900

**Published:** 2016-08-22

**Authors:** Yumiko Urano-Tashiro, Yukihiro Takahashi, Riyo Oguchi, Kiyoshi Konishi

In order to include a reference that was omitted from the originally published article, the authors add the following sentence to the beginning of the first paragraph under the subheading "Sugar-binding assay" in the Materials and Methods section: Sugar binding assay was performed as described previously [24, (Patel et al., 1999)].

The reference is: Patel N, Brinkman-Van der Linden EC, Altmann SW, Gish K, Balasubramanian S, Timans JC, et al. OB-BP1/Siglec-6. a leptin- and sialic acid-binding protein of the immunoglobulin superfamily. The Journal of Biological Chemistry. 1999; 274(32): 22729–38. doi: 10.1074/jbc.274.32.22729
